# High-resolution spin-echo Cardiac Diffusion-Weighted MRI with motion compensated Convex Optimized Diffusion Encoding (CODE)

**DOI:** 10.1186/1532-429X-18-S1-P26

**Published:** 2016-01-27

**Authors:** Eric Aliotta, Holden H Wu, Daniel B Ennis

**Affiliations:** 1 grid.19006.3e0000000096326718Radiology, UCLA, Los Angeles, CA USA; 2 grid.19006.3e0000000096326718Biomedical Physics, UCLA, Los Angeles, CA USA

## Background

Cardiac Diffusion Weighted MRI (cDWI) has the potential to characterize myocardial infarction (MI) without contrast. However, the clinical utility of cDWI has been limited by severe sensitivity to cardiac motion that manifests as signal dropouts which corrupt measures of myocardial diffusivity. This can be managed by carefully timing the diffusion encoding gradients (G_Diff_) to a quiescent diastolic phase, but this approach is burdensome and highly sensitive to heart-rate changes. More recently, motion compensated (MOCO) diffusion encoding gradients with nulled first (M_1_) and second (M_2_) moments have demonstrated good robustness to cardiac motion (Stoeck, MRM 2015, Nguyen, MRM 2013) but they necessarily increase the echo time (TE) compared to monopolar encoding (MONO), which reduces SNR and/or limits spatial resolution. We have developed a MOCO cDWI sequence that employs **Convex Optimized Diffusion Encoding (CODE)** to reduce bulk motion sensitivity and shorten TE compared to existing MOCO schemes.

## Methods

*G*_*Diff*_*Design*: CODE gradients were calculated using convex optimization to determine the M_1_ and M_2_ nulled G_Diff_ waveform that minimizes TE while conforming to hardware (G_Max_ = 80 mT/m and SR_Max_ = 50 T/m/s) and pulse sequence constraints. *Imaging*: Healthy volunteers (N = 5) were scanned on a 3.0T scanner (Siemens Prisma) after providing written informed consent for an IRB approved study. High resolution cDWI were acquired in the left ventricular (LV) short-axis with b = 350 s/mm^2^, 1.5 × 1.5 × 5.0 mm spatial resolution, 2x GRAPPA acceleration, three orthogonal diffusion encoding directions and three signal averages in a single 15-heartbeat breath hold. Both MONO (TE/TR = 67 ms/1R-R) and CODE encoding (TE/TR = 76 ms/1R-R) were acquired, but MOCO (TE = 94 ms) was not. All cDWI were acquired at eight subject-specific cardiac phases distributed across systole and diastole. *Reconstruction and Data Analysis*: Apparent diffusion coefficient (ADC) maps were reconstructed at each phase. Motion corrupted voxels were identified by ADC values exceeding 3.0 × 10^-3^mm^2^/s (the diffusivity of free water at 37°C, a thermodynamic upper bound for soft tissues) in the LV. The mean LV ADC and the percentage of motion corrupted LV voxels were then calculated at each phase. Statistical analyses were performed using t-tests with Holm-Sidak post hoc corrections.

## Results

**Figure 1:**
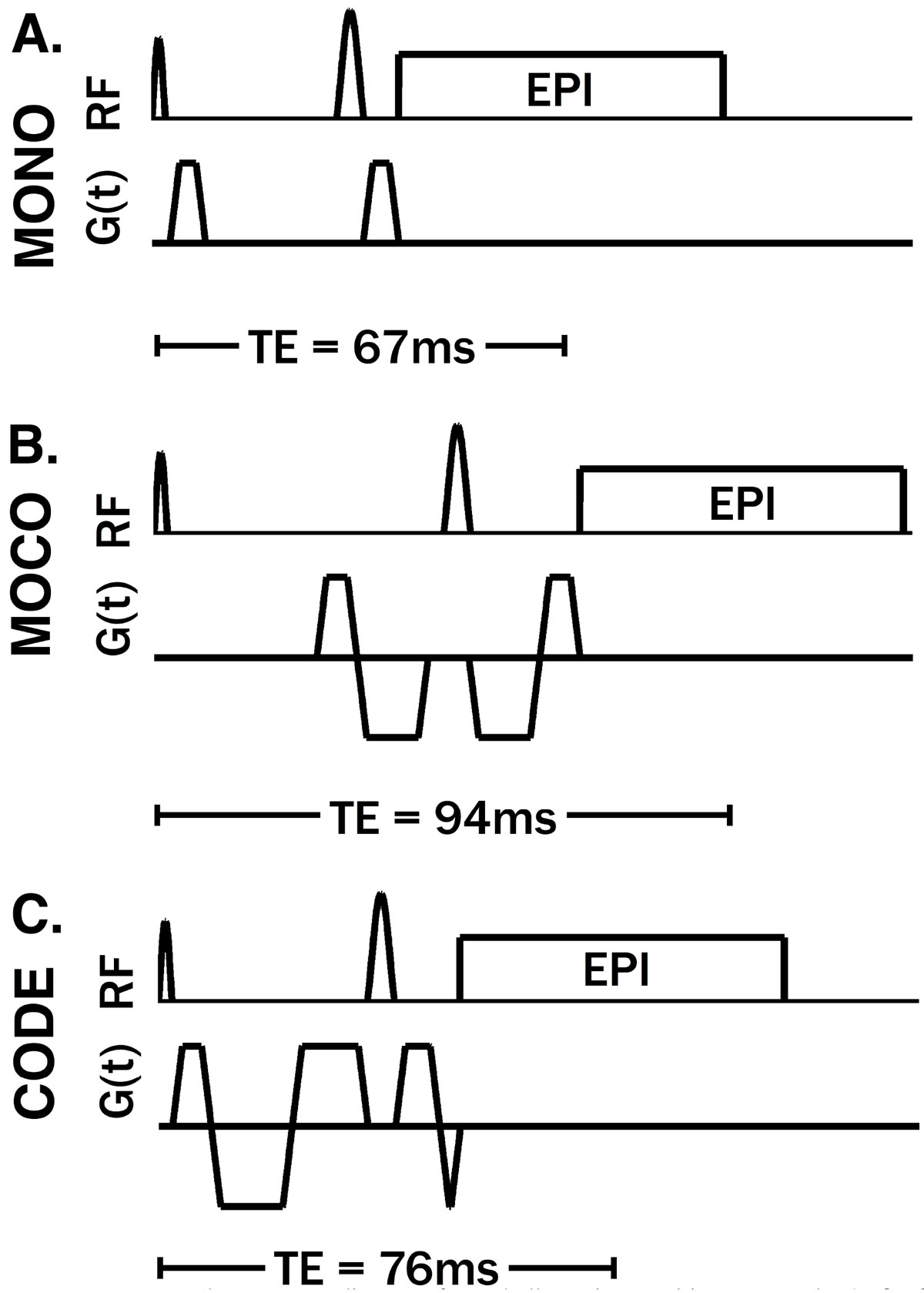
**cDWI pulse sequence diagrams for A. bulk-motion sensitive, monopolar (MONO) encoding; B. asymmetric bipolar motion compensated (MOCO) encoding and C. Convex Optimized Diffusion Encoding (CODE) encoding that is time-optimal, motion compensated (b = 350 s/mm**^**2**^ **and 1.5 × 1.5 mm in-plane resolution)**. MONO encoding (A) is fastest, but is not clinically reliable due to severe bulk motion sensitivity. MOCO (B) achieves bulk motion compensation, but requires a much longer TE than MONO (A) and CODE (C). CODE eliminates sequence dead time and improves SNR. CODE (TE = 76 ms) increases SNR by 49% compared to MOCO (TE = 94 ms), assuming myocardial T_2_ = 45 ms.

**Figure 2:**
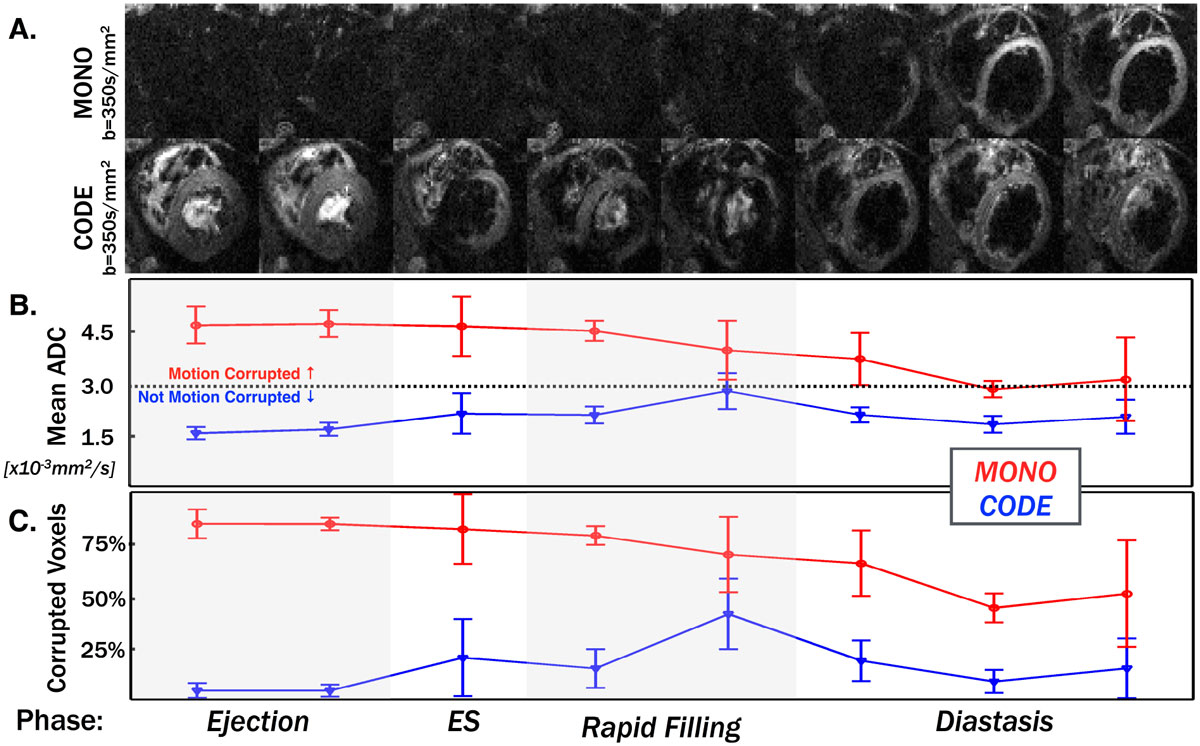
**A. Diffusion weighted images from a typical healthy volunteer acquired at eight different cardiac phases with MONO cDWI and CODE cDWI**. **B.** LV ADC values (Mean ± SD) and **C.** percentage ± SD of motion corrupted LV voxels (ADC>3.0 × 10^-3^mm^2^/s) for MONO cDWI and CODE cDWI encoding across the five volunteers. Motion corruption in MONO cDWI is highly subject dependent and varies greatly with cardiac phase. CODE cDWI is much less sensitive to bulk motion and is not dependent on precise sequence timing as shown by both the lower ADC measurements and lower percentage of motion corrupted voxels for all cardiac phases.

The TE for CODE (TE = 76 ms) is substantially shorter than asymmetric bipolar MOCO (Stoeck, MRM 2015) (TE = 94 ms) for 1.5 × 1.5 mm in plane resolution and b = 350 s/mm^2^, resulting in ~49% increase in SNR (Figure 1). *Mean ADC values were not significantly corrupted* (>3.0 × 10^-3^mm^2^/s) for 87.5% of phases with CODE (p < 0.01) and 0% of phases with MONO (p = N.S.) (Fig. 2B). CODE cDWI resulted in significantly *fewer motion corrupted voxels* than MONO in 87.5% of cardiac phases (p < 0.03) (Fig. 2C).

## Conclusions

CODE cDWI significantly improved robustness to cardiac motion compared to MONO cDWI. CODE cDWI also permits M_1_ and M_2_ moment nulling with a shorter TE than existing MOCO cDWI methods.

